# Dealing with care disruption in High and Intensive Care wards: From difficult patients to difficult situations

**DOI:** 10.1111/inm.12786

**Published:** 2020-09-16

**Authors:** Sylvia Gerritsen, Guy Widdershoven, Lia van der Ham, Laura van Melle, Martijn Kemper, Yolande Voskes

**Affiliations:** ^1^ Department of Medical Humanities Amsterdam University Medical Center VU University Medical Center Amsterdam The Netherlands; ^2^ Low Vision Research Ophthalmology Amsterdam University Medical Center VU University Medical Center Amsterdam The Netherlands; ^3^ Institute for Medical Ethics and History of Medicine Ruhr University Bochum Bochum Germany; ^4^ GGZ inGeest Amsterdam The Netherlands; ^5^ GGZ Breburg Tilburg The Netherlands; ^6^ Tranzo, Tilburg University Tilburg The Netherlands

**Keywords:** Inpatients, Intensive Care Units, Professional–Patient Relations, Psychiatry, Qualitative Research

## Abstract

High and Intensive Care is a relatively new care model in Dutch mental health care for clinical admissions. One of the goals is to keep the admission short. For some patients, this goal is not realized, which results in a long‐term admission. Often, this is experienced as a disruption. Disruptions in care processes are frequently defined in terms of patient characteristics. Yet, it may be that other factors play a role. The aim of this study is to gain better insight into the perceptions of care professionals of what is characteristic for disruptions at High and Intensive Care wards and how professionals can deal with these. Qualitative research was performed by means of semi‐structured interviews and a focus group with professionals. Results show that a focus on patient characteristics is too narrow and that other factors also play an important role. These factors include challenges in the relation between professionals and the patient, a divided team, and a lack of collaboration with ambulatory care. In order to deal with these factors, professionals should invest in the relationship with the patient, identify destructive team processes early, and improve communication with ambulatory care. It is recommended to develop a monitoring tool that includes all these factors. Another recommendation is to organize structured reflection on dilemmas experienced in care. In conclusion, this study shows the importance of going beyond patient characteristics in order to better understand, identify, and deal with disruption at High and Intensive Care wards.

## Introduction

Over the last years, new care models have been developed in Dutch mental health care. New approaches to ambulatory care have been implemented, such as Flexible Assertive Community Treatment (FACT) and Intensive Home Treatment (IHT) (Cornelis *et al*. 2018; Van Veldhuizen 2007). These models respond to policy transitions aimed at a reduction of the number of beds and prevention of the use of coercive measures in inpatient care settings, particularly seclusion. In the context of these changes, a new model for acute mental health care has been developed, called High and Intensive Care (HIC) (Van Melle *et al*. 2019). This model of care was developed in the Netherlands some years ago and is now implemented in nearly all mental health care organizations across the Netherlands.

The HIC model is based on best‐ and evidence‐based practices and the principle of stepped care (Van Melle *et al*. 2019). An admission is only indicated for patients in a severe psychiatric crisis for whom there is no other option. A short‐term admission is considered crucial in the HIC model as recovery takes place at home (Van Melle *et al*. 2019). Therefore, the intention is to limit the duration of admission to three weeks. If necessary, the admission can be extended twice for three weeks. For the majority of patients at HIC wards, the maximum criteria of three to nine weeks are realized. However, for a small patient population this appears to be challenging. For some patients, the duration of the admission runs into a couple of months, which implies a long‐term admission.

Long‐term admissions are often accompanied by disruption of the care process. In this context, disruption refers to disturbance at the ward related to the behaviour of a patient, experienced by care professionals and other patients. In these situations, care professionals do not know how to deal with the complex problems and needs of the patient. In mental health care, this complexity is often attributed to patients and their characteristics (Bos *et al*. 2012). In this context, the label ‘difficult patient’ is used by professionals. According to a study by Koekkoek *et al*. (2006, p. 796), patients are considered ‘difficult’ when they are: ‘(1) withdrawn and hard to reach, (2) demanding and claiming, (3) attention seeking and manipulating, or (4) aggressive and dangerous’. Patients diagnosed with schizophrenia, a personality disorder, a cognitive disorder, and alcohol or substance abuse were found more likely to be considered as difficult by psychiatrists (Sellers *et al*. 2012).

The focus on patient characteristics is questionable, as more factors may be relevant (Koekkoek *et al*. 2011a, 2011b,2011a, 2011b; Macdonald 2003). Kool *et al* (2014) emphasize that disruption involved problems in cooperation between care professionals and patient, caused by all those involved in the relationship. In particular, the role of the nurses is considered as important as they are in close contact with patients during the day. Therefore, it is important to gain a better understanding of what is involved in disruptions at HIC wards and the factors that play a role when professionals label a patient as difficult. The aim of this study is to gain better insight into the perceptions of care professionals of what is characteristic for disruptions at HIC wards and how professionals can deal with these.

## METHODS

### Design

A qualitative research approach was used to gain a better insight into the perceptions of care professionals of what is characteristic for disruptions at HIC wards and how professionals can deal with these. Qualitative research is an appropriate fit with the objectives of this study because it enables a better understanding of how professionals perceive disruption (Böhm 2004). The perceptions of psychiatrists, ward managers, nurses, a peer provider, and a trainer in de‐escalation techniques were explored in semi‐structured interviews and a focus group discussion (Flick 2018).

### Setting

This study was performed in a mental health care institution in the Netherlands. This organization provides specialized care to about ten thousand patients with severe and complex psychiatric problems, including care for patients with a severe, psychiatric crisis. The organization provides both ambulatory as inpatient care and has three HIC wards. All three HIC wards participated in the research; the wards are located in a different city but in the same region.

The HIC ward is a closed setting and provides the only possibility for an acute admission (Voskes *et al*. 2020). When there is a serious psychiatric crisis and there are no other options, patients in ambulatory care can be temporarily admitted to the HIC ward. During an admission, the referring ambulatory professional remains involved. Most often, the ambulatory care provider is working within the same organization. At the HIC ward, care is provided based on best practices. Examples include investing in contact with the patient, peer providers as part of the team, using a short‐term violence prediction instrument, and a comfort room (Abderhalden *et al*. 2008; Hedlund Lindberg *et al*. 2019; Simpson & House 2002; Van der Sande *et al*. 2011; Voskes *et al*. 2014). The HIC consists of two units, a High Care Unit and an Intensive Care Unit (Van Melle *et al*. 2019). In principle, patients stay at the High Care Unit. If needed, care can be intensified by providing one‐to‐one care in the Intensive Care (IC) Unit. The number of beds per HIC ward differs per organization. In terms of staffing, a ratio of 7 nurses on 20 beds is required; including other care professionals; this results in 20 professionals for each ward. In addition, the team consists of the following disciplines: psychiatrist, psychologist, nursing specialist, addiction specialist, (family) peer provider, activity counsellor, and a therapist.

### Participants

In total, sixteen professionals were interviewed. Perceptions regarding disruption related to long‐term admissions were explored from the perspective of professionals working at a HIC ward. From all three HIC wards of the participating organization, four professionals were interviewed; the ward manager and the psychiatrist, and two selected nurses. The nurses were selected by the ward manager because of their involvement in a recent situation of disruption. In addition, four professionals from outside the HIC wards who were involved in the provision of care for patients before and/or after an admission at a HIC ward were also interviewed. These included two nurses and one psychiatrist working in ambulatory care and a professional (project leader/manager) working at a clinic for intensive treatment. The interview participants consisted of a mix of eight women and eight men. The psychiatrists and ward managers were mostly middle‐aged; this was more diverse among the nurses. The work experience of the participants varied.

In order to recruit participants for the focus group discussion, a personal invitation was sent to all interviewed participants, of which four of them took part. Additionally, an invitation was sent to all professionals working at the three HIC wards within the organization, resulting in three additional participants. This was done in order to increase the number of participants and to bring a fresh perspective by not being interviewed before. In total, seven professionals participated in the focus group discussion. Participants in the focus group consisted of two ward managers, two nurses, one psychiatrist, one peer provider, and one trainer in de‐escalation techniques. Four of the participants were female, and three were male. Participants had a relatively long work experience and were of middle age.

The recruitment of participants was done by two of the authors (SG and MK). MK was working as a coordinator for the reduction of coercive measures within the organization and from this role familiar with the staff at these wards.

### Data collection

The interviews had a semi‐structured design and were audio‐recorded. The interviewer (SG) used an interview guide. Main topics in the interview guide focused on the characteristics of disruption at HIC wards and ways in which to deal with these. Part of the interview was to reflect on a recent case of disruption, brought in by the interviewees themselves. Interviews lasted ~45 min and were transcribed verbatim by the first author. One participant did not consent to the interview being audio‐recorded. In this case, data were collected in notes made by the interviewer.

The focus group aimed to identify and categorize characteristics of disruption at HIC wards and explore ways of dealing with these. The various characteristics were placed on post‐it notes by the participants, who were then invited to categorize them by making clusters of post‐its. In total, the focus group discussion lasted 90 min. The focus group discussion was audio‐recorded, transcribed verbatim, and supported by photographs of the categorization of the clusters of the characteristics.

The interviews and the focus group discussion were conducted in Dutch. The quotes of the participants were translated to English for the purpose of publication.

### Data analysis

The content of the interviews and focus group meeting was analysed using a sequence of open, axial, and selective coding (Boeije 2009; Corbin & Strauss 2014). To ensure the reliability and validation of the data, part of the data were analysed and interpreted by the co‐authors (Böhm 2004). Data analysis was supported by the use of a coding tree and the computer software program MAXQDA, version 10.

### Quality of data

A member check was performed for a correct interpretation of the interview participants’ vision (Steinke 2004). The interviewees checked the researcher's interpretations by reading the summary of their individual interview and providing feedback where necessary. Two participants made additions to the summary, which were included in the analysis.

### Ethical considerations

Information about the study was provided to the participants before the interviews and focus groups, after which informed consent was obtained. Pseudonyms were assigned to participants in order to ensure confidentiality.

## Results

This section offers an overview of the findings of this study. Firstly, a description is given of what is characteristic of disruptions, according to professionals. Secondly, the views of professionals on possibilities to cope with disruptions are presented.

## A. What is characteristic of disruptions?

Disruptions are characterized by a set of various aspects. Identified themes include patient characteristics, challenges in the relationships between professionals and the patient, a divided team, and lack of collaboration with ambulatory care.

### Patient characteristics

When talking about disruptions, professionals referred to the illness of the patient. The patient involved often seemed to have a combination of certain characteristics: a personality disorder, problems related to drugs (addiction), a low intelligence or/and a lacking awareness of their psychiatric condition, and/or a weak or problematic social network. In addition, aggressive or threatening behaviour by patients, both verbally and physically, was often mentioned. One of the nurses explained:

I find it hard to express myself about the intelligence of persons. Though it does suggest that these patients are a bit more faulty. I think they are the somewhat ‘the weakest link’. Their responses are often a bit more primary. Or they come from environments where aggression is normal anyway.

Although professionals often referred to patient characteristics as important factors in disruptions, the use of the label ‘difficult patient’ was regarded as one‐sided, undesirable, or even stigmatizing. The project leader of the clinic for intensive treatment said:

The moment you say the patient is disruptive and there is disruptive behaviour, and it doesn’t work out, the patient will be transferred. In other words, the patient is the problem. In my opinion the patient is not the problem but the situation, and I should say the relationship is the problem.

### Challenges in the relationship between professionals and the patient

According to participants, disruptions are often related to challenges in the interaction between professionals and the patient. Sometimes, professionals overestimate the patient’s abilities. Challenges may also be the result of not being able to explain the situation because of a patient’s lack of awareness of their psychiatric condition. Both overestimation of abilities and lack of awareness can give rise to a patient feeling misunderstood or hopeless, both of which result in an expression of frustration. This frustration can manifest itself in threats and displays of aggression. As one of the nurses put it:

The underlying problem is that these patients are often very sick, have no self‐awareness of their disease, or are in need of a long recovery period. These factors result in frustration among the patient […], which results in threats.

The impact of the threats and aggression affects professionals, as they feel intimidated or experience a lack of safety. Talking about this issue, a nurse said:

The last few times it was mainly about threats. The team doesn’t feel safe anymore as the patient made very intimidating and personal threats towards the nurses.

According to participants, a disruption is often associated with challenges in approaching patients, because of experienced fear by professionals. Participants also mentioned the complexity of distinguishing behaviour from illness. Behaviour may mistakenly be attributed to a patient's intention rather than resulting from the underlying disease. One of the team managers said:

Instead of seeing this woman as a patient, we start seeing her as an annoying person. At that moment, you are no longer professional.

According to professionals, providing care requires a personal approach by staff. Therefore, the relationship between professionals and patient may be damaged when professionals are affected by, for example, fear, powerlessness, or negative experiences. As a ward manager explained:

It's intense… the work requires the commitment of your own personality and being. You can't just press a button and turn off your own feelings. No, you're the (care), instrument in your work. This also makes it personal. When something happens in the provision of care, it becomes a personal thing between you and the patient. Even if it is caused by madness, fear or craving, this doesn't matter. It happens between two persons.

Professionals indicated that it can be difficult to restore mutual trust in the relationship with the patient, because the patient may lack the ability to reflect on the situation. A nurse said:

One of the patients constantly experiences so much frustration about the injustice we have caused him. He doesn't realize he's sick. He thinks we've been terrorizing him for five years.

### A divided team

Respondents associated disruptions with difficult team processes, especially a schism in the team. If a team is divided, this results in differences in both actions and vision. The team is no longer consistent in what is the right thing to do in the provision of care. For example, one of the nurses said:

Yes, at the moment we are divided as a team. Sometimes you see that there are different opinions, and we do not act, as discussion is ongoing. When the team is divided, you find yourself in a dangerous situation.

Disruption especially occurs when the team is divided in whether or not to set limits to patients. In case of a patient's challenging behaviour, some professionals do not set any boundaries out of fear that the patient may become angry or disturb the atmosphere at the ward. Other professionals do set limits. A divided team has a negative effect on patients and professionals. Patients experience a lack of clarity in what can or cannot be done, and consequently, the turmoil at the ward remains. Caregivers indicated that it is difficult that limits are not always set by colleagues. One of the ward managers explains:

You can try to keep a nice atmosphere all the time, though in certain situations you [as professional] need to be able to say that you do not tolerate it. Some people do [set limits] while others don’t. As a result, it becomes tough for the people who do [have to set limits].

In disruptive conditions, a team schism is not always noticed or noticed only at a late stage. Professionals indicated that this is challenging as staging an intervention in team processes at a later stage is more complex.

### Lack of collaboration with ambulatory care

According to the participants, a disruption can be characterized by challenges in the collaboration between the ward and the ambulatory professionals. Patients are sometimes admitted to the ward in an advanced crisis. These situations are complicated, as the patient's behaviour becomes more complex. In addition, ambulatory professionals are often burdened by the patient's advanced crisis, which makes them feel less able to provide care.

A disruption often is accompanied by a lack of continuity in care between ambulatory and clinical care. As a result, expectations do not match and misunderstandings arise between professionals. In addition, knowledge concerning the patient and positive and negative experiences are not sufficiently shared. Then, professionals at the ward feel that they are ‘reinventing the wheel’ when it comes to patient preferences and successes in approaching the patient. For example, a ward manager illustrated:

I think we, ourselves [the clinic], are not well organized and lack in the co‐operation with ambulatory care. How can you use inpatient care for only a short period of time? I think we only do this to some extent. We constantly reinvent the wheel every time someone goes to another place. Another team with the same system faces the same situations … at a certain moment the patient has seen all three the wards. We don’t learn enough from each other.

## B. How to deal with disruptions?

The interviewed professionals agreed that coping with disruptions requires a broader perspective, beyond focusing on the patient. The following aspects should be taken into account:

### Improving the relation between professionals and the patient

To deal with the challenges in the relationship and interactions with the patient, professionals should approach the patient in a professional way. Reflecting on the distinction between illness and a patient's behaviour can be helpful in this respect. The relationship between professionals and the patient is fostered when the professional has more understanding and insight into the patient's behaviour. In addition, continuous reflection and the sharing of feelings were envisioned to be necessary for early signalling and acting. As one ward manager reflected:

The moment the patient gets under your skin, it is already too late. So you have to make sure that the patient doesn’t get under the skin of one of the nurses. You should recognize early signals, given that everyone’s limit is different. Thereby, it is important that everyone dares to communicate this within the team. For instance, ‘I notice that I am scared to approach him’. It is professional to signal this and to do something with this feeling.

### Notice early signals in team processes

In order to deal with disruptions, professionals suggested that team processes should receive more attention. Early signals should be identified in order to prevent a team from becoming (more) divided. One of the nurses proposed working with a monitoring tool to make professionals more aware of team processes:

I think the focus should be more on us, on the team. A colleague has been proposing to work with a monitoring tool for the team for a couple of years now. They laugh at this, but he's actually absolutely right. What stage is the team in?

### Improving the collaboration with ambulatory care

Another way to deal with disruptions is to improve the collaboration with ambulatory professionals. HIC wards and ambulatory professionals should exchange knowledge, experiences, and best practices. Ambulatory professionals should receive more ownership and should remain involved in the provision of care. As a psychiatrist mentioned:

Ambulatory care needs to be expanded so that they [ambulatory professionals] really have time to provide treatment instead of just monitoring someone.

## Discussion

This study aimed to gain better insight into the perceptions of care professionals of what is characteristic for disruptions at High and Intensive Care wards and how professionals can deal with these. The findings of this study indicate that disruptions are not related to patient characteristics only, but also to the challenges in the relationship between the professionals and the patient, a divided team, and a lack of collaboration with ambulatory care. A change is needed from seeing disruption in terms of difficult patients to seeing it in terms of difficult situations.

Participants view patient characteristics as relevant. Patient characteristics that professionals experienced as potentially problematic correspond with the findings in the scientific literature. These characteristics include personality disorders, drug problems/dependency, low intelligence or/and a lacking awareness of their psychiatric condition, a weak or problematic social network, or/and aggressive or threatening behaviour (Fischer *et al*. 2019; Koekkoek *et al*. 2006; Koekkoek *et al*. 2011b; Sellers *et al*. 2012). The care professionals in our study do, however, consider the use of the label ‘difficult patient’ undesirable, as it stigmatizes and obliterates the influence of other important factors. These reflections are in line with previous research. According to Shattell (2004), the use of the label ‘difficult patient’ may cause professionals to distance themselves from the patient. Fischer *et al* (2019) emphasize that once a patient is labelled as difficult, it is hard to shed this label. Overall, our study suggests the need to focus on a wider range of factors that are relevant for disruptions in care processes.

The relationship between professionals and patient appears to be of great importance. In disruptions, there is often a high degree of aggression and threat. This has a major impact on professionals, especially nurses who may feel anxious and hesitant to act. This finding is in line with work by Voskes *et al* (2014) which notes both the importance of contact and a good relationship, and the challenge of making contact with an aggressive patient. Gabrielsson *et al*. (2016) mention that the moment professionals feel they cannot provide good care; this can result in frustration and distress. The negative feelings that professionals may experience, such as frustration and anxiety, in turn contribute to the labelling of a patient as difficult (Fischer *et al*. 2019). The findings of our study also show that there may be a lack of understanding on the part of the professional of the patient's behaviour. The challenging behaviour of a patient may be incorrectly attributed to a patient's intentions instead of to their illness. In addition, patients may lack awareness of their illness. Foster *et al* (2007) illustrated that fear and difficulties in understanding certain behaviours, such as aggression, may result in restraint and seclusion. Adler (2006) concluded that physical and emotional safety are needed for both professionals and patients. According to the findings in our study, not sharing feelings of insecurity in the team forms a risk for the onset of disruption.

This study shows that disruptions may be related to a schism within the team. This was also noted in other studies focusing on disruptions (Caruso *et al*. 2013; Fischer *et al*. 2019). Differing visions and ways of working clash, for example in setting limits. Setting limits may contribute to the enhancement of the therapeutic relationship and a reduction of aggression and coercive measures (Maguire *et al*. 2014). However, when a team is not consistent in setting limits, this can lead to distress among patients and increased aggression (Alexander 2006; Maguire *et al*. 2014). Conflicting perspectives and approaches in setting limits appear to exist among care providers. An approach proposed in this context is a common staff approach (Enarsson *et al*. 2007; Enarsson *et al*. 2008). This approach is used as a strategy to cope with difficult care situation, aiming to create unity and security among nurses (Enarsson *et al*. 2008). This approach focuses on agreement between staff regardless of the specific situation. Although unity in the staff is important, the situations investigated in our study show that unity can come under pressure, and that sticking to former agreements might result in putting the blame on the patient. Characteristic of difficult situations is that care professionals have different views, both regarding the patient and regarding their colleagues. Rather than trying to hold on to predefined agreements, professionals should explore each other’s underlying reasons and values behind certain actions, such as setting limits (Björkdahl *et al*. 2010; Enarsson *et al*. 2017; Vatne & Fagermoen 2007). This might create mutual understanding and finding a new way to jointly deal with the situation.

Within the HIC model, the referring ambulatory professional should remain in the lead (Van Melle *et al*. 2019). This study showed that disruptions are often associated with a lack of collaboration, involvement, and exchange of information between the ambulatory team and the HIC ward. Continuity of care, and provision of care, by the same professional is of great importance for people with a Severe Mental Illness (SMI; Crawford *et al*. 2004). Crawford *et al*. (2004) found that adequately coordinated care is challenged by staff shortages and limited time and resources. In order to deal with this, it is recommended to have clear methods of care coordination and the setting of clear agreements between team members (Crawford *et al*. 2004).

The findings of this research provide suggestions for improvement in how to deal with disruptions. The possibilities for improvement mentioned by professionals do not focus on the patient. An important reason for this is that patient factors often cannot be changed. Therefore, professionals themselves should change their approaches to dealing with disruption. A step forward could be to make a monitoring tool that describes how to identify potentially risky situations and how to act. This tool may contain (previous) experiences, signals, and best practices that may be relevant for the early identification of a disruption. It is desirable that the tool includes ways to monitor and if necessary improve the interaction between the professional and the patient, team processes, and the collaboration with ambulatory care.

Another practical implication in how to deal with disruption concerns the need for open communication and continuous reflection on experienced (negative) feelings. This can contribute to the early detection of disruption and increase mutual understanding within the team. Bowers *et al* (2010) also conclude that open communication among professionals decreases the risk on burnout and negative attitudes towards patients. A divide in the team may be prevented, or dealt with by reflecting specifically on motives and underlying values of professionals concerning certain actions or moral dilemmas. A relevant reflection method is Moral Case Deliberation. This entails a dialogue between professionals about the tensions they experience in care situations, structured by a facilitator, using a conversation method (Molewijk *et al*. 2008). Moral Case Deliberation is shown to have positive effects on teamwork, multidisciplinary cooperation, and attitudes towards the patient in mental health care by thinking from different perspectives, and understanding and communicating with each other (Hem *et al*. 2018).

This study has several strengths and limitations. A strength is the inclusion of various perspectives. The views and experiences of professionals working on various wards and having different backgrounds have been explored. In addition, the combination of interviews and a focus group meeting triangulated our findings and, as a result, contributed to a deeper understanding of the phenomenon of disruption, as both individual and collective experiences and reflections have been collected and explored. However, it is not clear whether the results of this study are transferable to all mental health care institutions with a HIC ward. There may be contextual differences, such as the region in which the institution is located. Additional research is warranted to establish whether these findings apply at a national level. Lastly, the perspective of the patient is absent in this study. Therefore, a follow‐up research is recommended to study disruptions from a patient perspective.

## Conclusion

In conclusion, this study shows that disruptions at HIC wards are not only related to patient factors, but also to the interaction between professionals and patient, team processes, and the collaboration with ambulatory care. Thus, it is important to shift the focus from difficult patients to difficult situations. In order to deal with disruption, professionals should identify and reflect on these factors. We propose to develop a monitoring tool for professionals in which the relevant factors are included. By using this tool, disruption at the HIC ward might be recognized earlier and dealt with more proactively and responsively.

## RELEVANCE FOR CLINICAL PRACTICE

For clinical practice, the findings of this study are relevant as they provide more insight into the factors involved in disruptions resulting in long‐term admissions and how to deal with them. The study shows the importance of focusing not only on patient factors, but also investing in the relationship with the patient, improving team processes, and fostering the relationship with ambulatory care. Practical suggestions for improvement are making a monitoring tool and organizing structured reflection on dilemmas experienced in care. This might particularly support nurses in dealing with these situations given their close contact with patients throughout the day, at HIC wards but also any other inpatient setting.
